# Biological and Synthetic Materials in Mandibular Reconstruction: Materials for Mandibular Reconstruction: Biological vs Synthetic

**DOI:** 10.31661/gmj.v13iSP1.3533

**Published:** 2024-12-08

**Authors:** Mehdi Abrishami, Maryam Jafari, Behnaz Dalvandi, Reza Dalvandi, Negar Sarrafan, Maryam Pourjebreil, Ali Alizadeh Noghani

**Affiliations:** ^1^ Department of Oral and Maxillofacial Surgery, Isfahan (Khorasgan) Branch, Islamic Azad University, Isfahan, Iran; ^2^ Department of General Surgery for trauma Shahid Beheshti University of Medical Sciences, Tehran, Iran; ^3^ Islamic Azad University, Tehran Medical Branch, Tehran, Iran; ^4^ Guilan University of Medical Sciences, Rasht, Iran; ^5^ Department of Oral and Maxillofacial Medicine, School of Dentistry, Urmia University of Medical Sciences, Urmia, Iran; ^6^ Department of Periodontitis, Dental Research Center, School of Dentistry, Hamedan University of Medical Sciences, Hamedan, Iran; ^7^ Sinus and Surgical Endoscopic Research Center, Department of Otorhinolaryngology, School of Medicine, Mashhad University of Medical Sciences, Mashhad, Iran

**Keywords:** Mandibular Reconstruction, Biological Materials, Synthetic Materials, Autografts, Allografts, Xenografts, Titanium, Polymeric Materials, Ceramic Materials, Bone Regeneration

## Abstract

Mandibular reconstruction is a critical surgical procedure necessary for restoring both function and aesthetics following trauma, tumor resection, or congenital defects. Over time, a variety of biological and synthetic materials have been developed to address the challenges of reconstructing the complex anatomy of the mandible. Biological materials, such as autografts, offer superior biocompatibility and osteogenic potential, but are limited by donor site morbidity and graft availability. Allografts and xenografts provide more accessible alternatives but are associated with higher risks of immune rejection and slower integration. In contrast, synthetic materials like titanium, PEEK (polyether ether ketone), and hydroxyapatite provide excellent mechanical strength and durability but often lack osteoinductive properties, requiring surface modifications to improve tissue integration.This review aims to provide a comprehensive analysis of the current materials used in mandibular reconstruction, comparing their biocompatibility, mechanical properties, osteoinductive potential, and clinical outcomes. Additionally, the review explores the growing role of composite materials that combine the strength of synthetics with the biological activity of natural tissues, as well as the advent of tissue engineering approaches that incorporate stem cell therapies and biomaterial scaffolds to promote bone regeneration. Emerging technologies such as 3D printing of custom-made implants and the application of nanotechnology for enhanced integration and infection control are also discussed as promising directions for future clinical applications. The findings highlight the need for continued research into optimizing biomaterial design and improving regenerative therapies to enhance patient-specific outcomes, reduce complications, and foster successful long-term integration of reconstructed mandibular structures. This review provides a roadmap for advancing both material science and clinical practice in the field of mandibular reconstruction.

## Introduction

Mandibular reconstruction has undergone significant advancements over the last century, evolving from rudimentary surgical techniques to highly specialized procedures integrating modern materials and technologies [1]. Over the decades, advances in surgical techniques and material sciences have significantly improved the outcomes of these procedures, particularly in cases involving trauma, tumor resection, or congenital defects [2]. Early efforts focused on autografts, where bone was taken from other parts of the patient’s body, but these procedures were often hindered by donor site morbidity and limited tissue availability [3]. The introduction of allografts and xenografts expanded options, while the development of synthetic materials like titanium and polymers offered new solutions for patients requiring structural support [4][5][6]. Despite these advancements, numerous challenges persist in mandibular reconstruction. Biological materials, such as autografts, while highly biocompatible and osteogenic, are limited by their availability and associated complications at the donor site [7]. Allografts and xenografts, although more accessible, carry higher risks of immune rejection, slower integration, and the potential for disease transmission [8][9]. On the other hand, synthetic materials such as metals, polymers, and ceramics provide excellent mechanical strength but lack the osteoinductive properties required for bone regeneration [10][11].

The objective of this review is to provide a comprehensive comparative analysis of the biological and synthetic materials used in mandibular reconstruction. This includes an examination of their biocompatibility, mechanical properties, osteoinductive potential, and clinical outcomes. Furthermore, the review will explore emerging tissue engineering approaches and innovative technologies such as 3D printing and nanotechnology, offering insights into how these advances are shaping the future of mandibular reconstruction.

## Biological Materials

Table-1 provides a clear overview of the various materials used in mandibular reconstruction, offering insight into their roles and challenges in clinical practice.

**Table:** Table1. Summarizes and Compares the Different Materials Utilized in Mandibular Reconstruction

**Category**	**Type**	**Source**	**Major Benefit **	**Major Limitation **
	Autografts	Patient’s bone (e.g., iliac crest, fibula)	High biocompatibility, osteogenic potential, no immune rejection	Donor site morbidity, limited availability of graft material
**Biological**	Allografts	Donor (cadaveric human bone)	Readily available in large quantities, no donor site morbidity	Risk of immune rejection, lower osteogenic potential
	Xenografts	Animal sources (e.g., bovine, porcine)	Readily available, can be osteoconductive	Risk of immune rejection, potential disease transmission
	Cell-Based Therapies	Patient-derived osteoblasts, chondrocytes	Promotes bone and cartilage regeneration, biologically active	High cost, experimental, potential cell survival challenges
**Tissue Engineering **	Stem Cells (MSCs, iPSCs)	Mesenchymal stem cells, induced pluripotent stem cells	High regenerative potential, promotes bone and tissue growth	Risk of tumorigenesis, immune response, experimental, expensive
	Biomaterials (Scaffolds)	Synthetic or natural biomaterials (e.g., collagen, PLGA)	Acts as a scaffold for tissue growth, customizable	Lack of mechanical strength may require additional bioactive agents
	Metals (Titanium, Stainless Steel)	Manufactured (titanium, alloys)	High mechanical strength, durability, good biocompatibility	Lack of osteoinductive properties, risk of stress shielding
**Synthetic**	Polymers (PMMA, PEEK)	Manufactured (synthetic polymers)	Lightweight, customizable, good mechanical properties	Poor osteoinduction, potential for soft tissue encapsulation
	Ceramics (Hydroxyapatite, Bioglass)	Synthetic or processed from minerals	Excellent biocompatibility, promotes bone integration	Brittle, lower mechanical strength, slow bone regeneration
**Composites**	Metal-Ceramic Composites	Combination of metals and ceramics	Combines strength with osteoconductivity, improved integration	Complex manufacturing, the potential for material degradation
	Polymer-Ceramic Composites	Combination of polymers and ceramics	Flexible, customizable, supports bone healing	Lower mechanical strength compared to metals, gradual resorption

### Bone Autograft

Autografts, particularly vascularized bone grafts such as the iliac crest graft, are commonly used in mandibular reconstruction due to their unique advantages. A major benefit is their high biocompatibility, as they originate from the patient's own body, minimizing the risk of immune rejection or graft failure [7][12]. This property allows autografts to integrate well with the recipient site, promoting natural bone healing and regeneration [7]. Vascularized bone grafts, such as the fibula or iliac crest, offer the added advantage of a blood supply, which is critical for the graft’s long-term survival and functionality.[13] The presence of a vascular system supports rapid integration, improves healing rates, and reduces the risk of infection or necrosis [12].

Furthermore, the iliac crest graft, being rich in both cancellous and cortical bone, provides a solid structure for large defect reconstruction while simultaneously ensuring osteogenic potential [14]. This versatility makes it an ideal choice for mandibular defects requiring both structural support and biological activity to enhance bone healing [14][15].

However, despite these advantages, autografts come with notable limitations. The most significant drawback is donor site morbidity, where patients experience pain, infection, or complications at the site from which the graft is harvested [14].

In cases where autografts are taken from the iliac crest, gait disturbances or functional impairment may also occur post-surgery [16]. Additionally, the amount of bone that can be harvested is limited, which constrains their use for larger defects [17]. The surgical procedure to harvest the graft increases the operative time and complexity, and in some cases, there may be delays in healing, especially if the patient has preexisting conditions that affect tissue regeneration [14][16][17].

### Bone Allografts

Allografts, which are grafts harvested from a donor of the same species (typically cadaveric donors), play an important role in mandibular reconstruction, especially when autografts are not a feasible option due to limitations in available tissue or concerns over donor site morbidity [4]. Allografts are primarily used in reconstructive surgery because they offer a ready source of bone material without the need for additional surgery on the patient, thus avoiding complications related to a second surgical site [18]. This ease of access and reduced operative time make them an appealing option in cases of extensive mandibular defects [18][19]. The primary benefit of allografts is their availability in larger quantities compared to autografts, which is advantageous when reconstructing large defects that require substantial bone material [19]. Moreover, allografts do not impose donor site morbidity on the patient, which is a significant advantage over autografts [4]. Allograft bone can also be processed to remove cells and proteins that might provoke an immune response, which increases its safety for use in transplantation. Another benefit is that allografts can be shaped and manipulated to fit the defect more easily during surgery, offering flexibility in their application [8].

However, despite these advantages, allografts carry certain risks [20]. The most prominent concern is the potential for immune rejection. Although processed to reduce immunogenic components, allografts still carry some risk of rejection, especially if the graft is not entirely decellularized [21]. Another major risk is the transmission of infectious diseases, although this risk is minimized through rigorous screening and sterilization protocols [20][21]. Additionally, allografts lack the intrinsic osteogenic potential that autografts provide [4]. Since they do not contain live cells, their ability to promote bone healing and integration is dependent on the host's regenerative capacity. This can lead to delayed or incomplete integration of the graft, which might result in graft failure over time [18][21].

Finally, osteoconductivity (the ability to act as a scaffold for new bone growth) in allografts is lower compared to autografts [4]. They primarily provide a structural framework but do not directly contribute to bone regeneration in the way that vascularized autografts do. As a result, allografts may be prone to slower healing times and can sometimes require supplemental procedures, such as the addition of growth factors or the combination with autografts to enhance bone regeneration [4][8].

### Xenografts

Xenografts, which are grafts derived from a different species (typically bovine or porcine), have been explored for use in mandibular reconstruction due to their potential availability and structural similarity to human bone [22]. xenografts are primarily applied as bone substitutes, providing a scaffold for the host’s cells to infiltrate and gradually remodel into functional bone [23]. They are particularly valuable when large amounts of graft material are required or when autografts and allografts are not viable options. Xenografts are processed extensively to remove cellular components, leaving behind a mineralized matrix that can promote osteoconduction, or the process of new bone growth along the graft material [5][24].

A major advantage of xenografts is their abundance and the fact that they eliminate the need for harvesting tissue from human donors, reducing the risk of donor site morbidity and making them easily accessible [5][22]. Furthermore, xenografts are often cheaper than autografts and allografts and can be prepared in different forms (e.g., granules, blocks) depending on the needs of the specific defect [5].

However, despite these benefits, challenges related to biocompatibility and immune responses significantly limit the broader use of xenografts [25].

The primary issue stems from the potential for immunogenicity, as tissues derived from non-human species naturally contain proteins and antigens that the human immune system may recognize as foreign [25]. Even after extensive processing to remove these immunogenic components, residual proteins or other molecular markers can still trigger an immune response [26]. This can lead to inflammation, graft rejection, or incomplete integration of the graft material with the host bone [27]. Another challenge is the risk of disease transmission from the donor species to humans, particularly when bovine or porcine materials are used [9][28]. While modern processing techniques, including sterilization and decellularization, aim to mitigate this risk, concerns about cross-species pathogen transmission remain [9].

## Tissue Engineering

Tissue engineering approaches have become increasingly promising in mandibular reconstruction as they aim to overcome the limitations of traditional graft materials by integrating advanced cell-based therapies, stem cells, and biomaterials [29][30]. These techniques focus on promoting regeneration by leveraging the body’s natural healing mechanisms in conjunction with engineered scaffolds and cellular components [30].

### Cell-based Therapies

Cell-based therapies involve the use of living cells to promote tissue regeneration and repair. In the context of mandibular reconstruction, osteogenic cells (cells capable of forming bone) are typically employed to enhance the regenerative capacity of the graft material. These cells are often seeded onto a scaffold (typically a biomaterial) and then implanted into the defect site, where they can proliferate and form new bone tissue [27].

The most commonly used cell type in mandibular reconstruction is osteoblasts the bone-forming cells responsible for producing the extracellular matrix and mineralizing the bone [31]. Chondrocytes, responsible for cartilage formation, and fibroblasts, which form connective tissue, are also sometimes utilized depending on the type of tissue to be regenerated [32]. The main challenge in cell-based therapies is ensuring that the transplanted cells survive, proliferate, and differentiate appropriately after implantation. To support this process, various biomaterials and bioreactors are employed to create the ideal environment for cell growth and differentiation [33].

### Stem Cells

Stem cells are particularly significant in tissue engineering because of their ability to differentiate into various cell types, including osteoblasts, chondrocytes, and endothelial cells. In mandibular reconstruction, the two primary types of stem cells used are mesenchymal stem cells (MSCs) and induced pluripotent stem cells (iPSCs) [12][34][35].

MSCs: These cells, often derived from bone marrow, adipose tissue, or dental pulp, are multipotent stem cells with the ability to differentiate into bone, cartilage, and other tissues relevant to mandibular repair [36][37]. MSCs can be isolated from the patient (autologous) or a donor (allogeneic), and they can be seeded onto scaffolds to promote bone regeneration [34]. Their regenerative potential is enhanced by the fact that they also produce various growth factors and cytokines that stimulate angiogenesis (formation of blood vessels) and reduce inflammation, creating a favorable environment for tissue repair [38][39].

IPSCs: These cells are generated by reprogramming somatic cells (such as skin cells) into a pluripotent state, allowing them to differentiate into any cell type, including osteoblasts [35]. iPSCs hold great promise due to their unlimited self-renewal and differentiation potential. iPSCs can be used to create patient-specific cell lines, reducing the risk of immune rejection [40]. However, challenges related to the safety and control of iPSC differentiation, particularly the risk of tumor formation, are significant hurdles that still need to be addressed [41].

### Biomaterials

Biomaterials form the foundation of tissue engineering scaffolds, which are crucial in providing structural support for cell attachment, proliferation, and differentiation. These scaffolds not only act as a template for new bone formation but also deliver cells, growth factors, and other bioactive molecules to the defect site. Major characteristics of ideal scaffolds include biocompatibility, biodegradability, mechanical strength, and porosity to allow for nutrient flow and vascularization [42][43].

Natural and Synthetic Biomaterials are used Commonly in mandibular reconstruction. Natural Materials like collagen, chitosan, and hydrogel are widely used due to their biocompatibility and ability to mimic the extracellular matrix of native tissues [44].

For example, collagen, derived from natural sources, is often used as a scaffold for bone regeneration due to its bioactivity and ability to promote cell attachment [45]. Chitosan, a derivative of chitin, also provides a biocompatible scaffold with osteoconductive properties [46].

Furthermore, Synthetic Biomaterials such as poly (lactic-co-glycolic acid) (PLGA), polycaprolactone (PCL), and polylactic acid (PLA) are frequently used to construct scaffolds [6]. These materials offer better control over the scaffold's mechanical properties and degradation rates. They can be tailored to degrade at a controlled pace, allowing the scaffold to support tissue formation while gradually resorbing once the new bone is formed [47]. Additionally, synthetic materials can be 3D-printed to create patient-specific scaffolds that perfectly match the geometry of the mandibular defect [48].

## Synthetic Materials

### Metals

Metals such as titanium and stainless steel have long been favored for their mechanical strength, durability, and biocompatibility [49]. Titanium, in particular, has emerged as the gold standard for metal-based implants due to its excellent properties in the context of bone reconstruction [50]. The major advantage of metal-based implants, especially titanium, is their remarkable mechanical strength, which is essential for withstanding the forces involved in mastication and other jaw functions. Clinical outcomes with metallic implants are pretty favorable, with high success in the restoration of mandibular function and aesthetics [42][49].The implants can turn out to be costly, as the implant processes and materials are highly advanced [42][51].

Titanium is also known for its biocompatibility, as it naturally forms a thin oxide layer on its surface when exposed to air, which helps prevent corrosion and promotes integration with surrounding bone tissue through osseointegration [50]. This property ensures a strong bond between the implant and the bone, reducing the risk of implant loosening over time [50]. Also, titanium is relatively lightweight, which minimizes patient discomfort and allows for easier handling during surgical procedures [52]. Clinical outcomes with titanium implants are generally excellent; the implants have high rates of success and prove to be durable in the long run [53]. Titanium implants are costly, and the material is expensive, so the price for an implant using that material would also be high enough [54]. On the other hand, stainless steel, though still used in some contexts, is generally less favored than titanium due to its susceptibility to corrosion and potential for causing localized tissue reactions [55]. While stainless steel is strong and more affordable than titanium, its corrosion resistance is inferior, particularly in the moist, biologically active environment of the mouth, which can lead to long-term degradation of the implant and inflammation of surrounding tissues [49][55].

However, despite these significant advantages, metal-based implants also come with limitations. The primary concern is their lack of biological activity. In opposition to biological materials, metals do not promote bone regeneration, meaning that they provide only structural support without contributing to the healing process [56]. This limitation becomes particularly evident in large defects where bone growth is required to fill gaps. Furthermore, although titanium integrates well with bone, it is not as effective in promoting soft tissue attachment, which can lead to complications such as tissue dehiscence or exposure to the implant [50][56].

Metal implants are also prone to infections, especially in cases where the surrounding soft tissue does not properly cover the implant. If infection occurs, it can be difficult to treat without removing the implant entirely [57]. Furthermore, while titanium implants are generally well tolerated, some patients may experience hypersensitivity or allergic reactions to metals, although this is relatively rare with titanium compared to other metals like nickel, which is often present in stainless steel [50][55][56].

### Polymeric Materials

Polymers such as PMMA (polymethyl methacrylate) and PEEK (polyether ether ketone) have become valuable materials in mandibular reconstruction due to their versatility, biocompatibility, and adaptability for both soft tissue and bone regeneration [58][59]. PMMA, commonly used in dental prosthetics and bone cement, provides a durable and customizable solution for filling bone defects [60]. Its primary advantage is its easy moldability during surgery, allowing for precise contouring to fit the patient's specific anatomical needs [58]. PMMA can act as a supportive scaffold, providing immediate structural stability in bone defects or fractures. However, it is bioinert, meaning it does not actively encourage bone regeneration or integrate with the surrounding tissue. As a result, it primarily serves as a space filler rather than promoting natural bone healing [60][61]. In addition, PMMA is prone to complications such as heat generation during polymerization, which can damage surrounding tissues, and susceptibility to infection due to its non-porous nature, making it challenging for soft tissue to integrate [61].

On the other hand, PEEK has gained popularity due to its superior biocompatibility, mechanical properties, and customization potential through advanced techniques like 3D printing. PEEK is a strong, lightweight polymer that can mimic the flexibility and mechanical behavior of bone more closely than PMMA [59][62]. This makes it particularly useful in patient-specific implants, especially in cases where complex mandibular defects need to be addressed with precise and durable solutions [59]. PEEK’s radiolucency (it does not interfere with imaging) is another advantage, making it easier to monitor post-operative healing through X-rays or CT scans [63].

Despite these benefits, polymers like PEEK and PMMA still face challenges in bone regeneration. While both materials provide structural support, neither of them is osteoconductive or osteoinductive [11]. They do not naturally support the growth of new bone or soft tissue integration, meaning they often require surface modifications or coatings with bioactive materials to enhance their interaction with the surrounding tissue [64]. Moreover, in the case of PEEK, soft tissue integration can be problematic as it does not bond naturally with tissue, potentially leading to fibrous encapsulation, where a fibrous tissue layer forms around the implant. This can weaken the integration and lead to implant mobility or failure over time [64][65]. Additionally, polymers can be subject to wear and tear over time, especially in the mechanically demanding environment of the mandible, where chewing forces are high. While PEEK is generally durable, long-term degradation or micro fracturing can occur, potentially leading to the need for revision surgeries [66][67].

## Ceramic Materials

Hydroxyapatite (HA) and bioglass are common ceramic materials increasingly used in mandible reconstruction due to their exceptional biocompatibility, potential for osseointegration, and, in some cases, osteoinductive properties [68][69].

HA, a naturally occurring mineral found in human bone, is particularly favored for its chemical similarity to bone tissue [68]. This property enables HA implants to bond directly with bone, promoting a strong and stable integration, which is crucial for long-term functionality in mandibular reconstructions [70]. Its biocompatibility is high, meaning it is well tolerated by the body with minimal risk of immune rejection or adverse inflammatory responses [70][71]. Similarly, bioglass has excellent biocompatibility and, when implanted, can form a HA-like layer on its surface, which enhances bonding with surrounding bone and tissue [71]. However, mechanical strength is a key area where ceramics generally lag behind metals. While HA and bioglass are strong enough to provide structural support in low-load areas, they are more brittle than metals like titanium [71]. This brittleness makes them susceptible to fractures under the high mechanical loads experienced in the mandible, particularly during mastication [71][72]. As a result, ceramics are often used as coatings on metal implants or in combination with other materials rather than as standalone solutions for weight-bearing applications [73].

One of the major advantages of ceramics specifically HA is their osteoinductive properties, which refers to their ability to promote the formation of new bone by stimulating osteoblast activity [68][69]. HA, in particular, encourages bone cells to migrate into the implant site and begin the process of bone regeneration [70]. Although this osteoinductivity is not as pronounced as that seen with autografts or stem cell-based approaches, it nonetheless makes HA a valuable material for encouraging natural bone healing around the implant [71]. Bioglass also possesses osteoinductive capabilities, and its ability to bond with both bone and soft tissue is particularly valuable in enhancing tissue integration and sealing the implant site [69].

## Composite Materials

Composite materials, which combine two or more distinct materials, are increasingly being utilized in mandibular reconstruction to capitalize on the advantages of each component while minimizing their limitations [73]. One common approach involves combining metals, such as titanium, with bioactive ceramics specifically HA [73][74]. Titanium provides the mechanical strength necessary to withstand the functional demands of the mandible, while HA contributes osteoconductive properties, promoting bone growth and integration [74]. This hybrid structure addresses a critical limitation of pure metal implants, which, although strong, do not naturally encourage bone regeneration. The ceramic coating acts as a bridge between the bone and the implant, improving osseointegration and reducing the risk of implant failure over time [73].

Another composite is the use of polymer-ceramic composites, such as PEEK combined with HA or bioactive glass [68]. In these composites, PEEK offers a lightweight, customizable scaffold with radiolucency, while the ceramic component enhances the biological activity, encouraging bone tissue ingrowth [75][76]. This combination mitigates the bioinert nature of polymers by introducing a material that interacts more favorably with biological tissues, improving bone regeneration and ensuring better integration with the surrounding tissue [76]. Also, these composite materials can be 3D-printed into patient-specific shapes, ensuring a more precise fit in complex mandibular defects [75].

Composite materials also address the challenge of soft tissue integration by incorporating biologically active coatings or growth factors [77]. For example, polymeric scaffolds can be coated with collagen or growth factor-releasing layers to enhance soft tissue attachment and reduce complications such as wound dehiscence or infection [65][73]. The growth factors help to stimulate cell migration and differentiation, accelerating the healing process and improving tissue regeneration around the implant [65]. Overall, the use of composite materials in mandibular reconstruction enables a more multifaceted approach to treatment by combining the structural advantages of metals or polymers with the biological functionality of ceramics or bioactive agents [77]. This results in improved mechanical performance, enhanced tissue healing, and better long-term outcomes compared to using single materials alone [78].

## Comparison of Biological and Synthetic Materials

**Table:** Table2. The Characteristics of Biological and Synthetic Materials used in Mandibular Reconstruction, Focusing on Biocompatibility, Mechanical Properties, Osteoinductive Potential, and Clinical Outcomes

**Characteristic**	**Biological Materials **	**Synthetic Materials **
**Biocompatibility**	High, especially for autografts (minimal immune response); allografts and xenografts may trigger immune reactions	Moderate to high; metals like titanium are highly biocompatible; some polymers (e.g., PEEK) can cause localized reactions
**Mechanical Properties **	Moderate; autografts provide good support but may lack strength for high-load areas; allografts are less mechanically robust	Excellent; metals like titanium offer high strength and durability; polymers like PEEK are flexible but less strong than metals
**Osteoinductive Potential **	High in autografts due to live cells and growth factors; allografts and xenografts have limited osteoinduction	Low to none; synthetic materials do not promote bone regeneration unless coated with bioactive substances like HA
**Clinical Outcomes **	High success with autografts (best integration, good long-term results); allografts/xenografts less predictable	High success in structural support and durability, especially for titanium; may require additional interventions for better integration
**Infection Risk **	Moderate to high, especially in allografts and xenografts due to possible contamination	Low, especially in titanium; infection risk increases if soft tissue coverage is poor
**Integration with Surrounding Tissue **	Excellent for autografts (both bone and soft tissue); lower for allografts and xenografts (slower integration)	Good for metals like titanium in bone; poor soft tissue integration for many synthetic materials without surface modifications
**Patient Outcomes **	Excellent functional outcomes with autografts, but donor site morbidity is a concern; allografts/xenografts may have variable success rates	Consistently good outcomes for structural support, but may face challenges with long-term integration and soft tissue healing

Both biological and synthetic materials have been used extensively, each with its strengths and limitations [77]. A comparative analysis of these materials based on biocompatibility, mechanical properties, osteoinductive potential, and clinical outcomes reveals distinct differences that influence their use in clinical practice. Table-2 highlights the strengths and limitations of both material types, illustrating how they perform across key factors relevant to mandibular reconstruction.

### Biocompatibility

Biocompatibility is generally higher for biological materials such as autografts, which are derived from the patient's own body. This makes them less likely to provoke an immune response or rejection [7][12]. Autografts, particularly vascularized bone grafts, have the added benefit of excellent tissue integration due to their natural cellular composition, making them highly compatible with the recipient site [13]. Allografts and xenografts, although derived from donors or different species, undergo extensive processing to improve biocompatibility, but they still pose a higher risk of immune rejection and adverse reactions compared to autografts [21][25].

In contrast, synthetic materials such as titanium, PEEK, and HA also demonstrate good biocompatibility, especially when appropriately processed [50][59][68]. Titanium is known for its remarkable biocompatibility, particularly its ability to form a stable oxide layer that prevents corrosion and promotes osseointegration [50]. However, some synthetic materials, such as PMMA, while generally well-tolerated, can provoke local inflammatory responses, especially if poorly integrated or improperly sterilized [60].

### Mechanical Properties

synthetic materials typically outperform biological options. Metals specifically titanium and polymers such as PEEK offer high mechanical strength and durability, making them particularly suited for load-bearing areas like the mandible [56][59]. Titanium's strength and resilience under mechanical stress ensure long-term stability in mandibular implants, while PEEK provides flexibility and a closer approximation to the mechanical behavior of bone [66]. Biological materials, on the other hand, such as autografts, tend to be less mechanically robust, especially when harvested from cancellous bone sources [79]. Vascularized autografts, however, can provide both structural support and biologic activity, making them an excellent option when both stability and regeneration are needed, though they may still not match the load-bearing capacity of titanium [13].

### Osteoinductive Potential

When it comes to osteoinductive potential, biological materials hold a clear advantage. Autografts, which contain living cells, growth factors, and natural bone matrix, are inherently osteoinductive, meaning they can stimulate new bone growth at the implantation site [4]. This is particularly advantageous in cases where bone regeneration is essential for healing [7]. Allografts and xenografts, while less osteoinductive due to processing that removes most cellular components, can still serve as osteoconductive scaffolds, allowing the host’s bone cells to grow into them [4].

In contrast, synthetic materials specifically titanium lack inherent osteoinductive properties. These materials primarily act as structural scaffolds, and bone regeneration around them depends entirely on the host's biological response [10][74]. To improve the osteoinductive potential of synthetic implants, coatings with bioactive materials such as HA or the addition of growth factors are often employed [10][73]. HA itself is osteoconductive and, to some extent, osteoinductive, promoting the attachment of osteoblasts and supporting bone ingrowth, although it is not as effective as autografts in driving active bone regeneration [73].

### Clinical Outcomes

Biological materials such as autografts tend to exhibit high success due to their ability to integrate well with surrounding tissue and promote natural bone healing. However, their success is often tempered by factors such as donor site morbidity, limited availability of graft material, and longer healing times [4]. Allografts have lower success rates compared to autografts, primarily due to risks of immune rejection, slower integration, and higher rates of complications such as resorption [54]. Xenografts generally have even lower success rates, largely due to their higher immunogenicity and challenges in long-term integration [22].

Synthetic materials, particularly titanium, have shown high clinical success rates, especially when durability and mechanical support are the primary considerations [42]. Their failure rates are generally low in terms of structural integrity, but complications can arise from poor soft tissue integration and the risk of stress shielding [74].

PEEK and other polymers also demonstrate favorable success rates, especially when used in conjunction with biological or bioactive coatings, though they can be prone to complications related to soft tissue encapsulation [64].

Infection rates tend to be higher with biological materials, especially in allografts and xenografts, where the risk of contamination, immune response, or improper sterilization during processing is greater. Autografts, while less prone to immune reactions, can still be susceptible to infection, particularly if the donor site is compromised [4].

On the other hand, Synthetic materials have lower infection rates due to their non-porous surfaces and resistance to bacterial colonization, especially in metals [57]. However, infection can still occur, particularly if the implant is exposed to the oral environment due to wound dehiscence or tissue breakdown [80].

Moreover, biological materials generally outperform synthetics in tissue integration. Autografts, especially vascularized bone grafts, integrate well with both the hard and soft tissues, fostering a seamless transition between graft and native bone [13]. Synthetic implants, while stable, often struggle with soft tissue integration, which can lead to complications like wound breakdown, exposure of the implant, or the formation of a fibrous capsule around the material [65].

Finally, patient outcomes tend to be more favorable with autografts due to their regenerative capabilities, but the trade-offs include longer recovery periods and the risk of complications at the donor site [16][23]. Synthetic materials, particularly metals such as titanium and PEEK, offer faster recovery with reliable structural support, though they may require more frequent follow-up to address issues related to long-term integration and soft tissue complications [81].

## Innovations and Emerging Technologies

The future of mandibular reconstruction is being shaped by innovations specifically 3D printing, custom-made implants, and nanotechnology, which are transforming the field by improving precision, functionality, and patient outcomes [48][62][69]. 3D printing allows for the creation of patient-specific implants that precisely match a patient's unique anatomy, reducing complications and improving surgical outcomes [67]. These implants, often made from materials such as PEEK, can be custom-designed based on detailed imaging data from CT scans [63]. This not only enhances the fit and mechanical function of the implant but also reduces operative time and post-surgery complications [59]. Nanotechnology is also playing an increasingly important role, with nanostructured surfaces improving biocompatibility and osteointegration by enhancing cell attachment and tissue regeneration [34]. Moreover, nanoparticles can be used to deliver bioactive molecules directly to the reconstruction site, offering the potential for targeted healing and infection control [82].

Another significant area of ongoing research in mandibular reconstruction involves stem cell-based therapies. MSCs are being explored for their potential to regenerate both bone and soft tissue in mandibular defects [34]. These stem cells, which can differentiate into osteoblasts and other relevant cell types, are typically delivered via scaffolds made from biocompatible materials, encouraging the growth of new tissue at the defect site [34][38]. IPSCs, which can be reprogrammed from a patient’s cells, offer a promising future in personalized regenerative medicine, minimizing immune rejection and maximizing tissue integration [40]. However, challenges such as controlling differentiation and preventing tumorigenesis still need to be overcome before these approaches can be widely adopted in clinical practice [41].

Emerging experimental therapies also include bioprinting and the use of exosomes [83]. Bioprinting uses cell-laden bio-inks to create layered tissue constructs that can potentially mimic both bone and soft tissue, offering the potential for fully regenerative solutions in complex mandibular defects [83][84]. Exosomes, small vesicles released by stem cells, are being explored as an alternative to traditional stem cell therapies [85]. These vesicles contain growth factors and signaling molecules that can stimulate tissue regeneration without the risks associated with direct stem cell implantation, such as immune rejection or tumor growth [83]. These cutting-edge approaches promise to revolutionize mandibular reconstruction by offering more precise, biologically active, and patient-specific solutions.

## Conclusion

The mandibular reconstruction relies on a diverse array of biological and synthetic materials, each offering distinct advantages and limitations. Autografts remain the gold standard due to their superior biocompatibility and osteogenic potential, but their use is constrained by donor site morbidity and limited availability. Allografts and xenografts provide larger volumes but come with increased risks of immune rejection and slower integration. Synthetic materials like titanium, PEEK, and ceramics offer excellent mechanical strength and durability but lack inherent osteoinductive properties, requiring bioactive coatings or hybrid materials to improve tissue integration. Composite materials, which combine the strengths of different material types, present a promising solution to these challenges, offering improved mechanical and biological performance.

Looking ahead, tissue engineering approaches including cell-based therapies, stem cell-based regeneration, and biomaterial scaffolds hold significant promise for advancing the field. Innovations like 3D printing of custom-made implants and the application of nanotechnology are driving the development of more personalized and biologically active reconstruction techniques. These innovations aim to enhance implant fit, improve tissue regeneration, and reduce complications associated with current methods.

Future research should focus on optimizing biomaterial design, improving stem cell delivery systems, and advancing nanotechnology to create more effective and integrated solutions for mandibular reconstruction. Furthermore, clinical studies should aim to refine patient-specific approaches and assess the long-term outcomes of emerging technologies, ensuring that new materials and methods not only meet functional demands but also promote successful tissue regeneration and integration.

## Conflict of Interest

None.
